# Synthesis and evolution of physicochemical properties of new pharmaceutically active ionic liquids – tetracainium salicylate and tetracainium ibuprofenate†

**DOI:** 10.1039/d2ra04711j

**Published:** 2022-09-21

**Authors:** Maksim Rapaić, Jovana Panić, Branislava Teofilović, Nevena Grujić-Letić, Slobodan Gadžurić, Milan Vraneš

**Affiliations:** Faculty of Sciences, Department of Chemistry, Biochemistry and Environmental Protection, University of Novi Sad Trg Dositeja Obradovića 3 Novi Sad 21000 Serbia jovanap@dh.uns.ac.rs; Faculty of Medicine, University of Novi Sad Hajduk Veljkova 3 Novi Sad 21000 Serbia

## Abstract

Tetracainium salicylate and tetracainium ibuprofenate were synthesized as active pharmaceutical ingredient ionic liquids (API-ILs). These ILs represent a combination of a drug for local anaesthesia (tetracaine) and nonsteroidal anti-inflammatory drugs (salicylic acid and ibuprofen). After IL synthesis, spectroscopic investigations were performed using infrared and nuclear magnetic resonance spectroscopy to confirm their structures. Differential scanning calorimetry and thermogravimetric analysis determined the obtained thermal behaviour of the ionic liquids. Experimental density, viscosity, and electrical conductivity measurements were performed in a wide temperature range to understand the interactions occurring in the obtained pharmaceutically active ionic liquids. All experimental values were well-fitted by the empirical equations. According to the theoretical calculations, weaker interactions of tetracaine with ibuprofenate than with salicylate are found, ascribed to the decreasing molecular symmetry, weakened hydrogen bonding, and increasing steric hindrance of ibuprofenate's alkyl chain.

## Abstract 2

Evolution of physicochemical parameters such as thermal stability, density, viscosity and electrical conductivity at various temperatures for two pharmaceutically active ionic liquids – tetracainium salicylate and tetracainium ibuprofenate.

## Graphical abstract

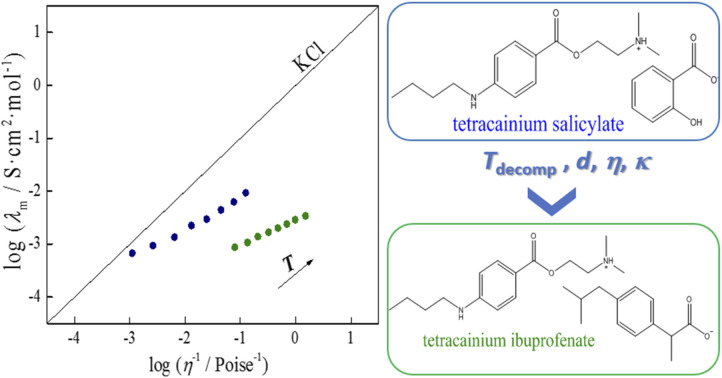

## Introduction

Solid-state salt formation is the current primary approach to improving active pharmaceutical ingredient (API) properties. Also, ionic liquid formation with appropriate, non-toxic counterions could provide another option and greater flexibility in salt selection. Here, a novel active pharmaceutical ingredient ionic liquid (API-IL) concept is presented, suggesting use of traditional drugs in the form of ionic liquids.^1–6^ The field of ionic liquids covers salts that melt below 100 °C and are usually composed of large asymmetric cations and organic/inorganic anions. They possess a unique and convenient advantage through their tunability. The synthesis of APIs in the form of ionic liquids, with a cation or anion as a pharmacologically active substance, completely eliminates the problem of their low solubility in water, prevents the possibility of building polymorphous forms and enables the achievement of a much higher concentration of active components compared to solid formulations. With the correct selection of cations and anions, it is possible to adjust their physical and chemical properties to improve bioavailability, facilitate transport through the cell membrane, and so on.^1,2^ The possibility of making APIs ionic liquids containing biologically active cations and anions should be emphasised by obtaining a single compound with a dual function. For example, pyridoxinium-ascorbate, which contains biologically available and easily applicable forms of vitamin B6 (pyridoxine) and vitamin C (ascorbic acid), is a synthesized ionic liquid.^5^ Also, the procainium salicylate and procainium ibuprofenate ionic liquid, a combination of a local anaesthetic and an anti-inflammatory drug, has already shown 4 to 10-fold higher solubility than the initial drugs.^6^

The data on physicochemical properties of ionic liquids are essential for theoretical research and industrial application. Thus, this work analysed thermal stability, density, viscosity and electrical conductivity at various temperatures for two newly synthesized ILs. Tetracainium salicylate and tetracainium ibuprofenate were chosen as API-ILs, as a combination of a drug for local anaesthesia (tetracaine) and a nonsteroidal anti-inflammatory drug (salicylic acid and ibuprofen).

## Experimental section

### The materials and general procedure of tetracaine-based ionic liquids preparation

All applied chemicals were used without purification. Tetracaine (CAS number: 94-24-6, mass fraction purity ≥0.98), tetracaine hydrochloride (CAS number: 136-47-0, mass fraction purity ≥0.98), and ibuprofen (CAS number: 15687-27-1, mass fraction purity ≥0.98) were obtained from TCI chemicals (Tokyo, Japan). Salicylic acid (CAS number: 69-72-7, mass fraction purity ≥0.99) and methanol (HPLC grade, mass fraction purity ≥0.99) were obtained from Sigma-Aldrich (Steinheim, Germany).

Tetracainium salicylate ([Tet][Sal]) and tetracainium ibuprofenate ([Tet][Ibp]) were prepared according to the earlier reported procedure.^6^

**Fig. 1:**
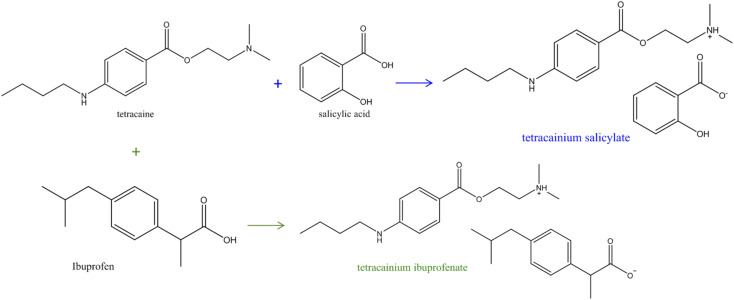
Synthesis scheme of tetracainium salicylate and tetracainium ibuprofenate ionic liquids.

An appropriate amount of tetracaine with the equimolar amount (1 : 1) of salicylic acid/ibuprofen was measured, dissolved in methanol, and stirred overnight at room temperature (Fig. 1). The solvent was evaporated using a rotational evaporator at a temperature of 353.15 K. Both ionic liquids remained yellow liquids at room temperature with no tendency for crystallisation during the work. For the structure confirmations and the purity determination of the synthesized ILs, the IR, and NMR spectra were recorded, and the assigned structures of these compounds were confirmed (Fig. S1–S4 in the ESI†). All characteristic IR and NMR peaks were found. From Fig. S1b,† the disappearance of the C=O stretching of the carboxyl group of salicylic acid at 1660 cm^−1^ was obvious while a new band appeared at about 1602 cm^−1^ indicating protonation of the carboxyl group occurred in tetracainium salicylate. IR spectra in Fig. S2b† show the shifting in wavenumber of the C=O stretching vibration from 1719 to 1701 cm^−1^ for the ibuprofen carboxylic acid group, together with decreased peak intensity. This change of C=O stretching vibration in the tetracainium ibuprofenate indicates the possible formation of the hydrogen bond between the carboxylic acid group and the cation. The purity of 98.6% for synthesized [Tet][Sal] and 98.4% for [Tet][Ibp] was determined using the earlier reported HPLC method with the regression coefficient (*R*^2^) for tetracine calibration curve of *R*^2^ = 0.9998 (Fig. S5†).^6^

### Infrared spectra

Infrared spectra were recorded as neat samples from (4000 to 650) cm^−1^ on a Thermo Nicolet Nexus 670 spectrometer fitted with a Universal ATR Sampling Accessory. The measurements were performed with a total of 60 scans, at *T* = 298.15 K, and a spectral resolution of 2 cm^−1^. The software package omnic version 6.2 was used in the data acquisition and spectral analysis.

### Nuclear magnetic resonance spectra

The NMR spectra of tetracainium salicylate and tetracainium ibuprofenate were recorded in DMSO-d6 at *T* = 298.15 K on a Bruker Advance III 400 MHz spectrometer. Tetramethylsilane was used as an accepted internal standard for calibrating chemical shifts for ^1^H and ^13^C. ^1^H homodecoupling and the 2D COSY method were used routinely to assign obtained NMR spectra. ^13^C NMR spectra were assigned by the selective decoupling technique.

### Thermal characterisation

Thermogravimetric (TG) analysis was performed using simultaneous TG/DSC thermal analyser SDT Q600 (TA Instruments, USA). The sample (≈3.0 mg) was placed in an open platinum pan. The measurements were carried out in an argon atmosphere (flow rate 50 cm^3^ min^−1^) up to 450 °C with a heating rate of 20 °C min^−1^. Differential scanning calorimetric (DSC) measurements were performed by differential thermal analyser DSC Q20 (TA Instruments, USA) with a heating rate of 20 °C min^−1^ in a nitrogen atmosphere. The samples were cooled to −80 °C, held at that temperature for 20 min, and then heated to 100 °C.

### Density measurements

The vibrating tube Rudolph Research Analytical DDM 2911 densimeter was used for density measurements. The instrument was thermostated within ±0.01 K, and viscosity was automatically corrected. Before each series of measures, calibration of the device was performed at the atmospheric pressure (*p* = 0.1 MPa) using ambient air and bi–distilled ultra-pure water in the temperature range from (293.15 to 353.15) K. Each experimental density value is the average of at least five measurements at selected temperatures. Repeated experimental measurements showed repeatability within 0.01%, and an average value is presented in this paper. The measurement was conducted in the temperature range from (293.15 to 353.15) K for [Tet][Ibp], and from (308.15 to 353.15) K for [Tet][Sal] at atmospheric pressure (*p* = 0.1 MPa). The standard uncertainty of determining the density is less than 3.0 × 10^−3^ g cm^−3^.

### Viscosity measurements

The viscosity of investigated ionic liquids was measured using a Brookfield Viscometer DV II + Pro thermostated within ±0.01 K and filled with about 16 cm^3^ of pure liquid. The viscosity of investigated ionic liquids was measured in the temperature range from (293.15 to 353.15) K, and the revolution per minute (RPM) was set to obtain a proper torque. The spindle type SC4-18 was used for [Tet][Ibp] in the whole temperature range, while [Tet][Sal] was used only in the temperature range from (318.15 to 353.15) K. For measuring viscosity of [Tet][Sal] at lower temperatures, the spindle type LV-4(64) was used due to high viscosity. A viscometer cell was protected from moisture with the manufacturer's compartment and calibrated using the liquids of different viscosities purchased from the manufacturer. Presented experimental values are the mean of five measurements, and the measurement relative standard uncertainty was about 2.0%.

### Electrical conductivity

Electrical conductivity measurements of pure ionic liquids were determined by a conductivity meter Jenco 3107 using a DC signal in a Pyrex cell with platinum electrodes. The experimental temperature ranged from (318.15 to 353.15) K. The relative standard uncertainty for electrical conductivity was less than 1.5%. All the obtained experimental values represent the mean of three measurements.

## Results and discussion

### Thermal stability

**Fig. 2:**
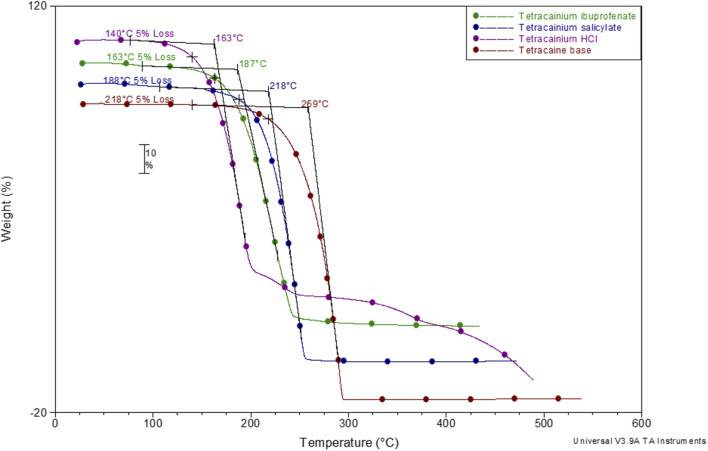
Thermogravimetric curves of examined compounds.

To establish the suitability of the ILs for use at elevated temperatures, their thermal stability was studied by thermogravimetric analysis (TGA). In common with other ILs, API-Ls do not boil at high temperatures but show upper thermal stability limits.^7^ TGA data for both new ILs, as well as for tetracaine base and tetracaine hydrochloride, are presented in Fig. 2. Fig. 2 shows the percentage mass loss as a function of temperature for the ILs heated to a maximum temperature of 450 °C at a rate of 20 °C min^−1^. The decomposition onset temperatures (*T*_onset_) and the decomposition temperatures corresponding to 5% mass loss (*T*_5% onset_) are determined from TGA curves. The gathered data indicate that the new ILs are stable at approximately *T*_onset_ = 187 °C with *T*_5% onset_ = 163 °C for tetracainium ibuprofenate, and *T*_onset_ = 218 °C with *T*_5% onset_ = 188 °C for the tetracainium salicylate. Comparing the thermal activity of the tetracaine base with tetracaine hydrochloride and new ILs, it can be concluded that converting the neutral base tetracaine into ionic compounds decreases its stability. Within the ionic compounds, stability depends on the anion's nature. Namely, thermal stability decrease in order: tetracainium salicylate > tetracainium ibuprofenate > tetracainium hydrochloride. First, it can be observed that tetracaine forms more thermal stable salts with organic anions than with inorganic chloride. This can be an effect of the packaging and additional π–π interactions between cation and anions, salicylate and ibuprofenate. Further, a smaller salicylate anion is closest to tetracaine cation, while enhanced alkyl chain length of ibuprofenate leads to the loose packing of ions and decreases the efficiency of π–π interactions in tetracainium ibuprofenate structure.

**Fig. 3:**
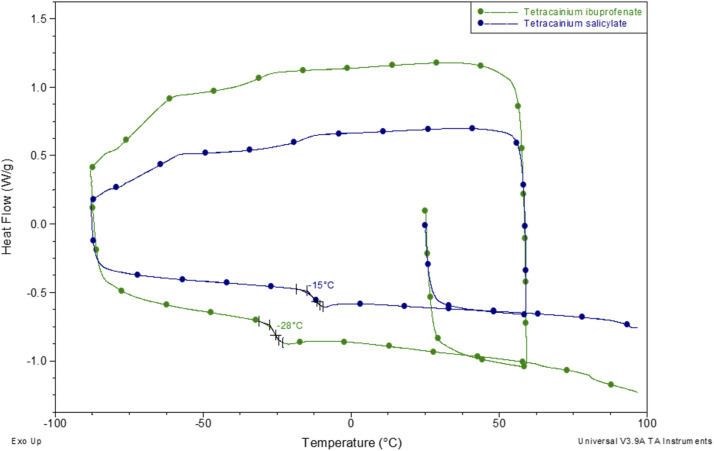
Differential scanning calorimetry curves of tetracainium ibuprofenate and tetracainium salicylate.

Further, phase transition temperatures of newly synthesized ionic liquids using differential scanning calorimetry (DSC) were investigated (Fig. 3). The glass transition temperatures (*T*_g_) values of tetracainium ibuprofenate at *T*_g_ = −28 °C and tetracainium salicylate at *T*_g_ = −15 °C can be seen from DSC curves, representing characteristic phase transitions for ionic liquids. The tetracainium ibuprofenate and tetracainium salicylate API-Ls showed no freezing or melting point.

### Density results

**Fig. 4:**
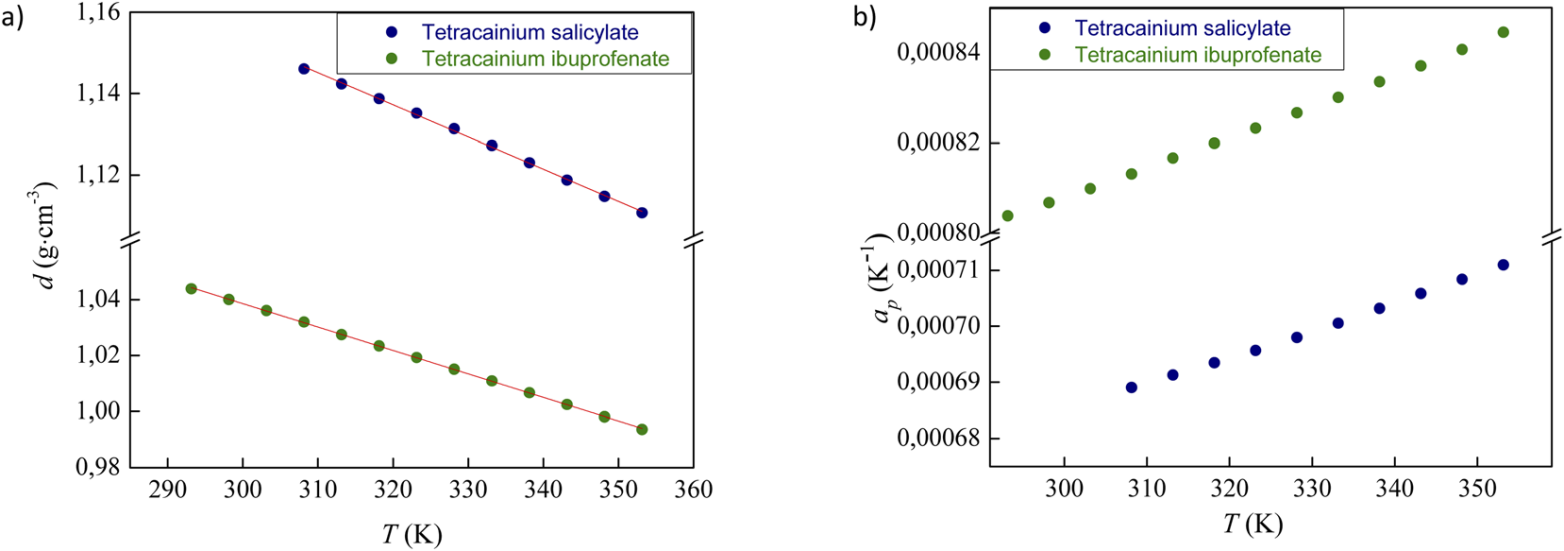
Variation of (a) density and (b) thermal expansion coefficients of ILs with temperature.

The experimental data for the density (*d*) of neat ILs tetracainium ibuprofenate and tetracainium salicylate are reported in Table S1† and presented in Fig. 4a.

The ions in ionic liquid structure tend to get closer and pack into the crystal lattice. As expected, density depends on the anion size, which can be seen from higher obtained density values of tetracainium salicylate than tetracainium ibuprofenate at all the examined temperatures. It can be concluded from the obtained results that salicylate as a smaller ion gets much closer to tetracaine and induce a better package, which leads to the higher density values of tetracainium salicylate than tetracainium ibuprofenate.

From the density data, the thermal expansion coefficients (*α*_p_) representing the rate at which the cation moves away from the anion with rising temperature were calculated (eqn (1)). The obtained data are reported in Table S1† and presented in Fig. 4b. Higher values of tetracainium ibuprofenate than tetracainium salicylate also indicate better packaging in [Tet][Sal] structure. It reflects that the extension of anion size weakens the interaction between ions, which promotes the expansion of ILs.1
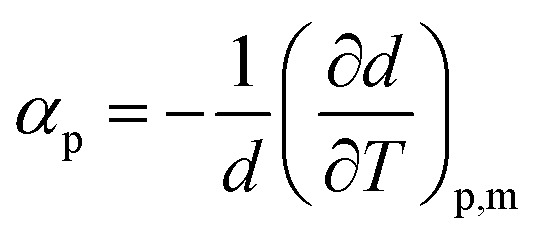


**Fig. 5:**
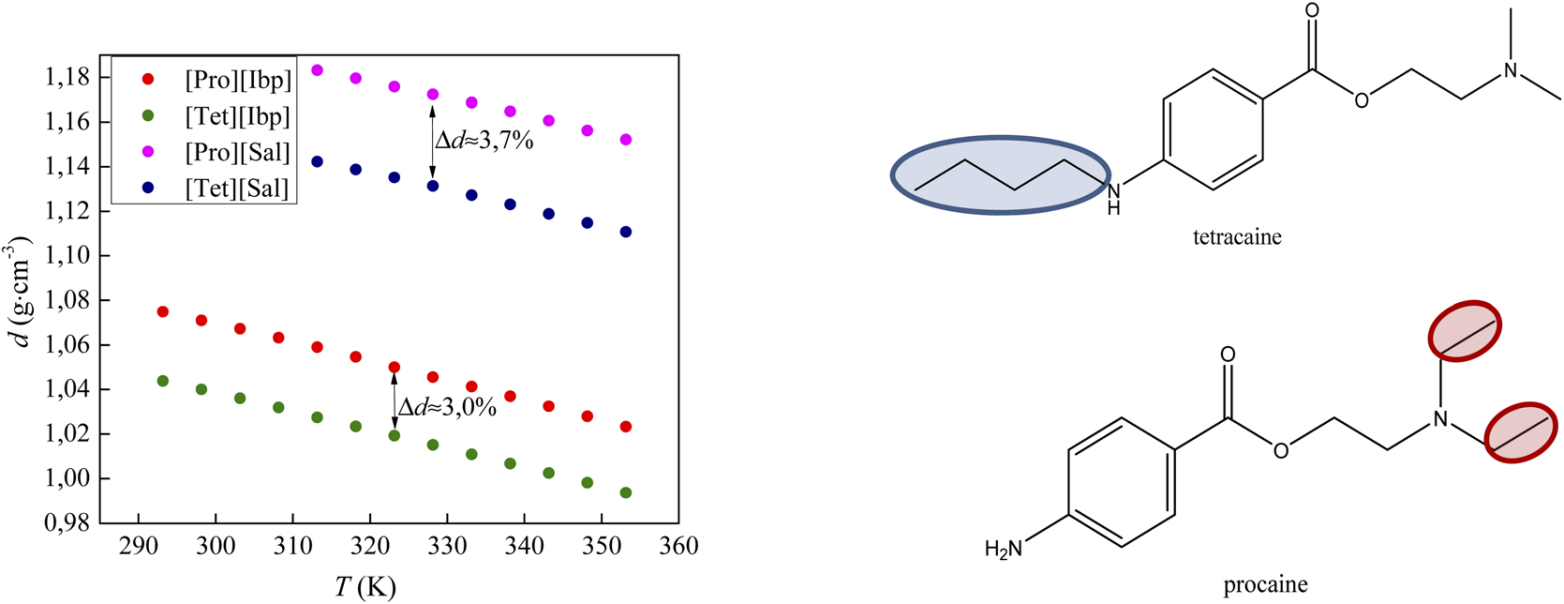
Comparison of densities values of tetracaine-based ionic liquid with procaine-based ionic liquids.

Fig. 5 compares the density results obtained in this paper with the density values from our previous work for procaine-based ionic liquids – procainium salicylate and procainium ibuprofenate.^6^ The figure shows that the values of tetracaine-based ionic liquids are lower and that the differences are 3.0% for IL with ibuprofen as an anion, and 3.7% in the case of salicylates. The lower densities probably stem from the additional butyl chain on the amino group of the benzene ring, which decrease molecular symmetry and sterically hinders the approach to the benzene ring and reduces the possibility of establishing stronger π–π interactions.

### Viscosity results

**Fig. 6:**
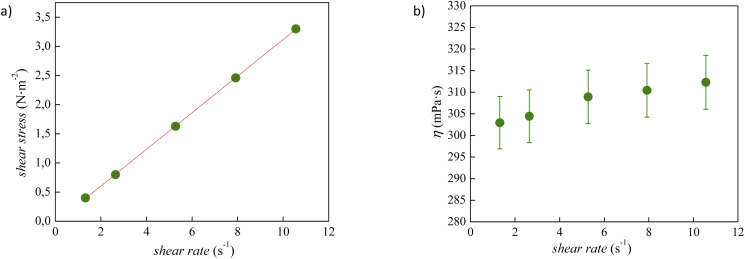
(a) Flow curve, showing shear stress as a function of shear rate for pure tetracainium salicylate; (b) changes of viscosity values at 353.15 K with shear rate along with error bars.

**Fig. 7:**
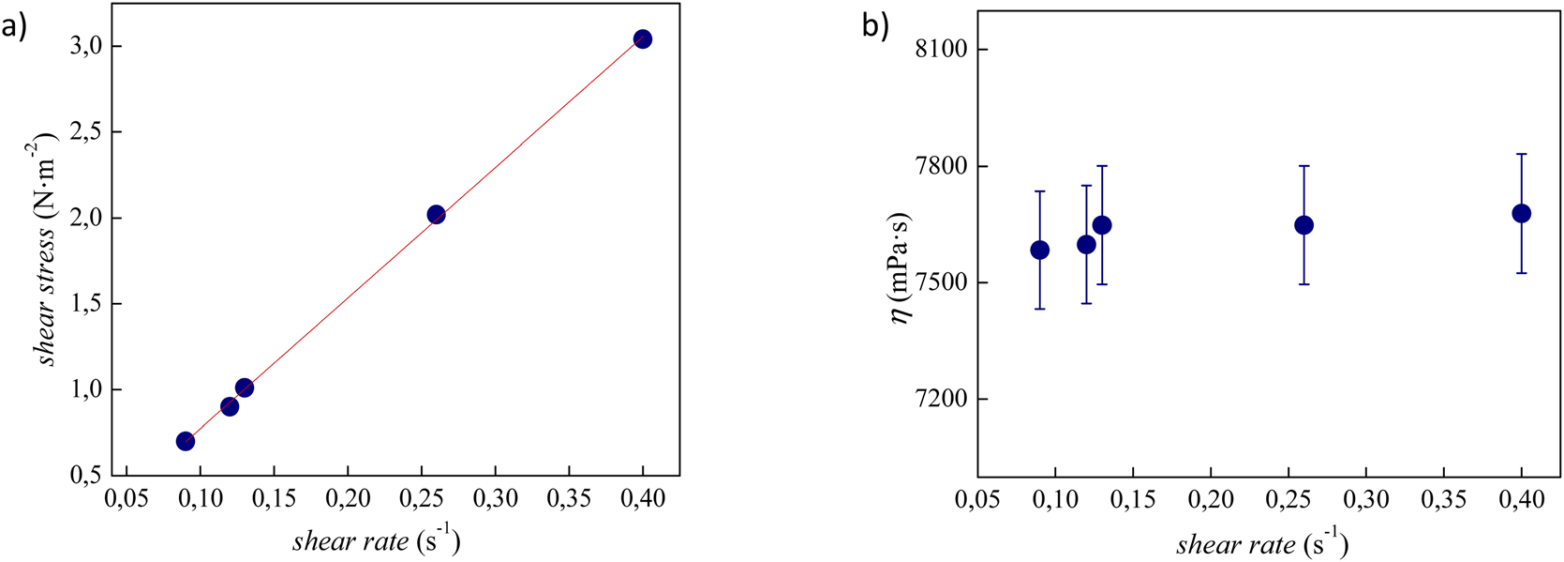
(a) Flow curve, showing shear stress as a function of shear rate for pure tetracainium ibuprofenate; (b) changes of viscosity values at 353.15 K with shear rate along with error bars.

The experimental viscosity data (*η*) of tetracainium salicylate and tetracainium ibuprofenate are listed in Table S2.† Firstly, it was observed, that for the studied ILs, the viscosity remains constant with increasing shear rates, as presented in Fig. 6 and 7. A linear relationship between the shear stress and the shear rate indicates a Newtonian behaviour of all studied ILs.

**Fig. 8:**
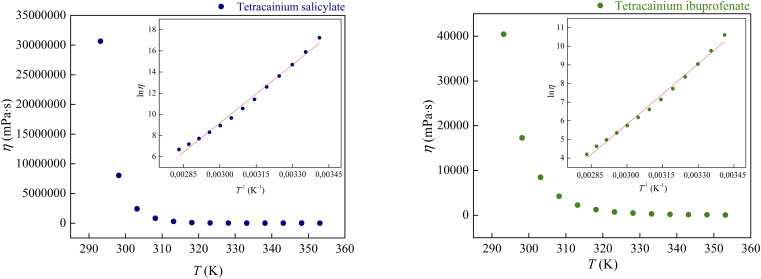
The viscosity dependence on temperature with incorporated Arrhenius plot.

Fig. 8 demonstrates the correlation between viscosity and temperature for these ILs. The presented results indicate that the viscosity of tetracainium ibuprofenate is 1000 times lower than the viscosity of tetracainium salicylate. This phenomenon is probably ascribed to decreased molecular symmetry, weaker interactions between cation and anion, and increased steric hindrance in the tetracainium ibuprofenate structure. Additionally, if one compares the viscosity values of these tetracaine-based ionic liquids with ionic liquids containing procaine as a cation, a significant difference can be observed.^6^ Namely, for ionic liquids with salicylate as an anion, the obtained values are *η* = 38 291 mPa s for tetracainium salicylate, while for procainium salicylate amounts *η* = 137 670 mPa s at *T* = 323.15 K. Ibuprofen ionic liquids have viscosity values of *η* = 743.3 mPa s for tetracainium ibuprofenate while for procainium ibuprofenate amounts *η* = 3153 mPa s at *T* = 323.15 K.

It can be concluded that the viscosity values for procaine-based ionic liquids are about four times more viscous than tetracaine-based ionic liquids. Additional butyl chain bounded to the amino group of the tetracaine benzene ring decreases molecular symmetry and additionally sterically hinders the approach to the benzene ring and reduces the possibility of establishing stronger π–π interactions and thus reduces viscosity.

The experimental data of viscosity (*η*) at different temperatures (*T*) were fitted by the linearized Arrhenius equation:

2 ln(*η*) = ln *C* + *E*_a_/*RT*

where *C* is the adjustable parameter, *R* is the universal gas constant, and *E*_a_ represents activation energy of the viscous flow. The activation energy of the viscous flow is a crucial property that controls the temperature dependence of the viscosity. The values of *E*_a_ = 152.28 kJ mol^−1^ for tetracainium salicylate and *E*_a_ = 89.82 kJ mol^−1^ for tetracainium ibuprofenate are obtained. The results indicate that the ibuprofenate ionic liquid required lower energy to initiate viscous flow.

### Electrical conductivity results

In Table S3† are given the experimental electrical conductivity data (*κ*) of tetracainium salicylate and tetracainium ibuprofenate. Then the electrical conductivity values are used to calculate molar conductivity (*λ*_m_) as *λ*_m_ = *κM*/*d* where *M* represents molar mass and *d* is the density of the pure ionic liquid (Table S3†).

**Fig. 9:**
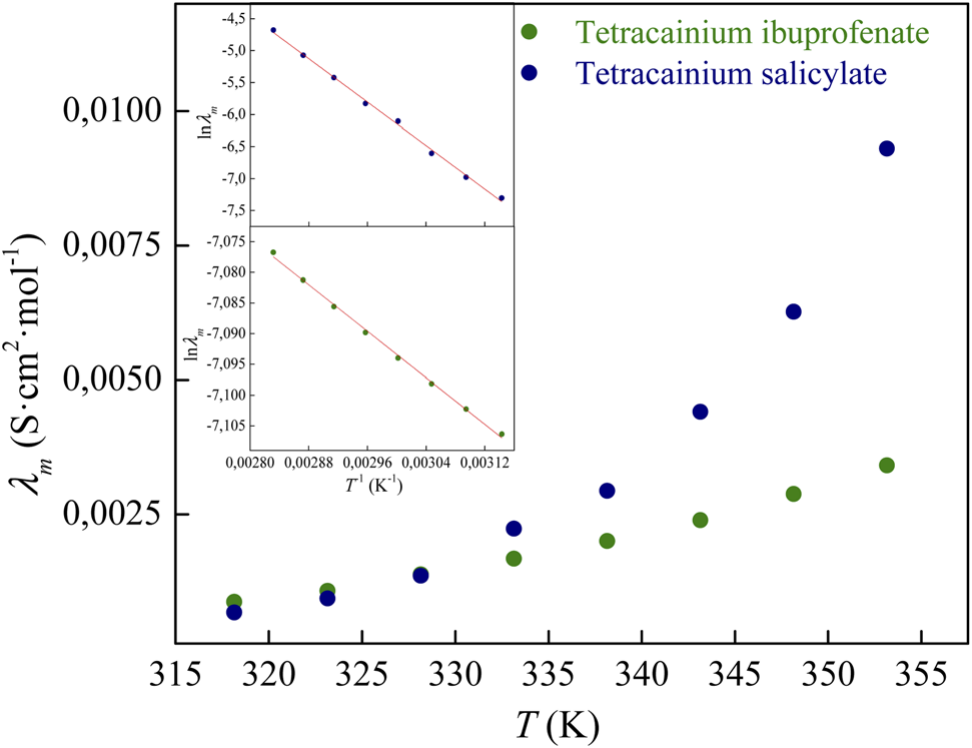
The electrical conductivity dependence on temperature for synthesized ionic liquids with incorporated Arrhenius plot.

The temperature dependency of molar conductivity is illustrated in Fig. 9. The values of molar conductivity (*λ*_m_) at experimental temperatures (*T*) were fitted by the linearized Arrhenius equation.^8^3
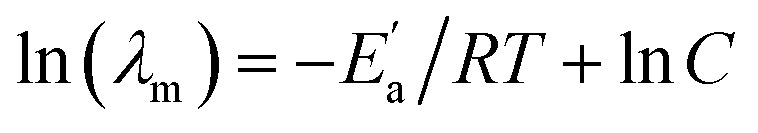
where 
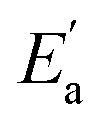
 represents conductivity activation energy. The values of 
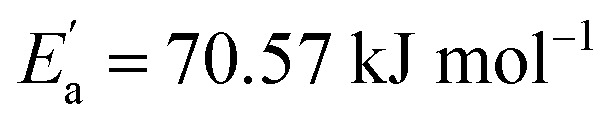
 for tetracainium salicylate and 
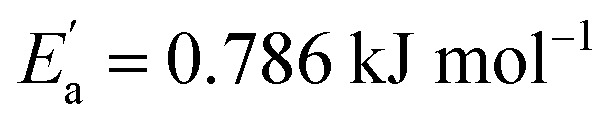
 for tetracainium ibuprofenate are obtained. A higher 
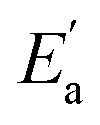
 indicates that the molar conductivity of the tetracainium salicylate ionic liquid is more sensitive to temperature changes. Also, from Fig. 9, it can be seen that up to the temperature *T* = 328.15 K, the molar conductivity values are slightly higher for tetracainium ibuprofenate. Further, as temperature rise, a much more significant difference in the molar conductivity values are achieved, with higher values for tetracainium salicylate than for tetracainium ibuprofenate. This could be explained by strong interactions between ions in tetracainum salicylate at lower temperatures. On the other hand, at higher temperatures, the interactions weaken, and the ions move more freely. Hence, the smaller salicylate moves away from the tetracaine cation faster and easier, leading to higher molar conductivity values. The anion of ibuprofen is larger with decreased molecular symmetry, the alkyl chain length hinders the ion mobility and charge transfer so consequently, temperature rise increases somewhat the molar conductivity values.

**Fig. 10:**
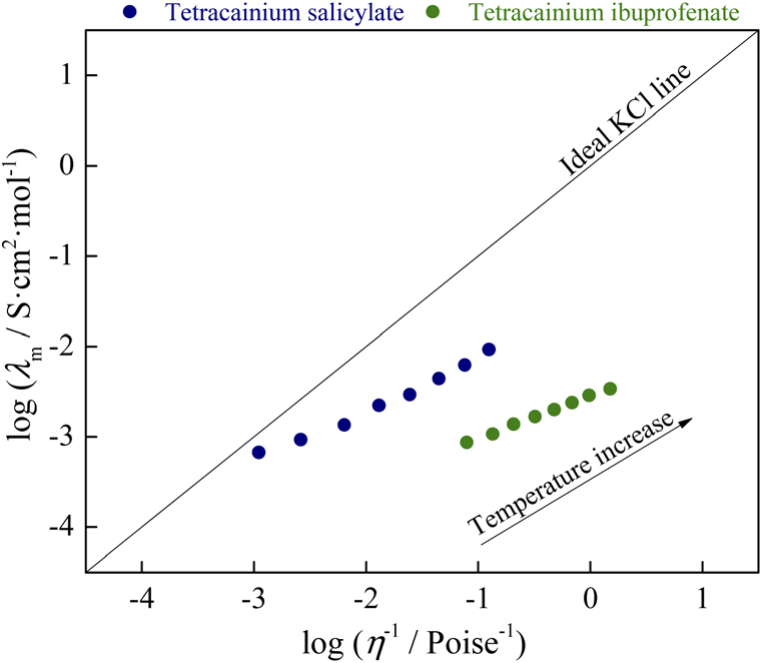
Walden plot.

The molar conductivity and viscosity obtained in this study were correlated by the following linear fitting of the Walden equation:

4 log *λ*_m_ = log *C* + *α* log *η*^−1^

where *α* is the slope of the line in the Walden plot, which reflects the ions' decoupling and *C* is constant. The temperature-dependent Walden plot is presented in Fig. 10. The straight line in the Walden plot, drawn for reference, represents the theoretical behaviour of 0.01 M KCl aqueous solution, where the ions are known to be entirely dissociated and to have equal mobility.^9^

From Fig. 10, it can be seen that both ionic liquids lie below the ideal KCl line. In aqueous solutions of electrolytes, such as the reference 0.01 M KCl, the increase in molar conductivity is due exclusively to a decrease in the viscosity of the solution. Walden rule explains the deviation in systems where the ions are not completely dissociated. Namely, molar conductivity is also affected by the degree of ion dissociation among viscosity. The more significant the deviation from the ideal behavior of KCl is consequence lower the degree of ion dissociation. Our systems have the opposite situation: an ionic liquid with a higher viscosity (tetracainium salicylate) has a higher molar conductivity. This is a consequence of numerous additional interactions between ions (H-bonds, π–π interactions, van der Waals interactions), which, among viscosity, significantly determine the molar conductivity of ionic liquids. The classification of examined ionic liquids can be made by the vertical distance measured from the KCl ideal line to the point of ionic liquid, also called Δ*W* (deviation below ideal KCl line):

5 Δ*W* = log *η*^−1^ − log *λ*_m_

Based on the obtained Δ*W* value, ionic liquids can be divided into three groups.^10–12^ The first group covers the range of 0.1 < Δ*W* < 0.5, and it is made up of almost independently mobile ions and can be classified as good ionic liquids. The second group's range is 0.5 < Δ*W* < 1.0 and is named “poor” ionic liquids. In this group, H-bonds and other specific interactions between ions in the pure state are more pronounced, leading to a significant reduction of ions mobility. The third group represents the liquids that are at least an order of magnitude below the ideal line, also described as liquid ion pairs or “subionic liquids” (Δ*W* > 1.0). In these liquids, ion conductivity is substantially less than expected based on their viscosity. The simplest example is the ion pair, which is electrically neutral and does not contribute to the measured conductivity.

From the Δ*W* data, % ionicity of ionic liquids can be calculated as follows:

6 % Ionicity = 10^−Δ*W*^ × 100%

The obtained values of Δ*W* and % ionicity are summarized in Table 1. The obtained values of deviation and ionicity indicate that complete proton transfer did not occur in the structure of tetracainium ibuprofenate and that predominantly ionic pairs are present. On the other hand, the structural organization of ions in tetracainium salicylate changes through all three groups with temperature. In previous work with procaine-based ionic liquids, the value of % ionicity at *T* = 328.15 K was higher than tetracaine-based ionic liquids with % ionicity of 64.5% for procainium salicylate, while for procainium ibuprofenate obtained value was 3.34%.^6^ The reason for a lower % ionicity of ibuprofen based ionic liquids is due to ibuprofen with p*K*_a_ = 5.3 is approximately one hundredfold weaker acid than salicylic acid with p*K*_a_ = 2.9. Additionally, between the ions of ibuprofen and tetracaine, there is certainly a greater number of non-covalent interactions that further complicate and limit the dissociation of ionic liquid. In general conclusion, the presence of the butyl group at the tetracaine benzene ring significantly influences the organisation of ions in ionic liquids structure and its interaction strength.

**Table 1:** Calculated deviation (Δ*W*) and ionicity for [Tet][Sal] and [Tet][Ibp] at different temperatures

*T* (K)	Δ*W*		% Ionicity	
	[Tet][Sal]	[Tet][Ibp]	[Tet][Sal]	[Tet][Ibp]
318.15	0.21	1.96	61.0	1.10
323.15	0.45	2.10	35.7	0.80
328.15	0.67	2.18	21.2	0.67
333.15	0.77	2.28	17.1	0.52
338.15	0.92	2.38	12.0	0.42
343.15	1.00	2.46	9.91	0.35
348.15	1.08	2.53	8.27	0.30
353.15	1.13	2.64	7.47	0.23

## Conclusions

Within this research, two ionic liquids, tetracainium salicylate and tetracainium ibuprofenate were successfully synthesized by the acid–base neutralization method. IR and NMR spectroscopy was applied to confirm the structure of the synthesized ionic liquids. Thermal analysis showed that the tested ionic liquids were thermally more stable than the hydrochloride salt of tetracaine. The melting point and freezing point were not obtained under the applied conditions. The density, viscosity and electrical conductivity of pure ionic liquids over a wider temperature range were further measured. The thermal coefficient of expansion was calculated from the density values, indicating stronger cation–anion interactions in the tetracainium salicylate structure. The obtained data on viscosity and electrical conductivity are well-fitted with the Arrhenius equation, from which the values of activation energies are obtained. All results indicate stronger interactions in the structure of tetracainium salicylate, which is a consequence of the better geometric fit of the smaller salicylate ion than ibuprofenate. Namely, an alkyl chain in the ibuprofenate structure leads to decreasing molecular symmetry, weakened hydrogen bonding, and increased steric hindrance. Also, used Walden equation showed better ionization of tetracainium salicylate than tetracainium ibuprofenate, which can be explained by the smaller size of salicylate, which moves away from cation more freely and easily.

## Author contributions

Maksim Rapaić: methodlogy, writing and editing. Jovana Panić: analysis, investigation, writing and editing. Branislava Teofilović and Nevena Grujić-Letić, synthesis and characterization. Slobodan Gadžurić, software and data curation. Milan Vraneš, editing and review.

## Conflicts of interest

There are no conflicts to declare.

## Supplementary Material

RA-012-D2RA04711J-s001

## References

[cit1] Pedro S. N., Freire C. S. R., Silvestre A. J. D., Freire M. G. (2020). Int. J. Mol. Sci..

[cit2] Bica K., Rodríguez H., Gurau G., Cojocaru O. A., Riisager A., Fehrmannd R., Rogers R. D. (2012). Chem. Commun..

[cit3] Egorova K. S., Gordeev E. G., Ananikov V. P. (2017). Chem. Rev..

[cit4] Stoimenovski J., MacFarlane D. R., Bica K., Rogers R. D. (2010). Pharm. Res..

[cit5] Sugibayashi K., Yoshida Y., Suzuki R., Yoshizawa K., Mori K., Itakura S., Takayama K., Todo H. (2020). Pharmaceutics.

[cit6] Panić J., Tot A., Drid P., Gadžurić S., Vraneš M. (2021). Eur. J. Pharm. Sci..

[cit7] Yu J., Wheelhouse R. T., Honey M. A., Karodia N. (2020). J. Mol. Liq..

[cit8] Arrhenius S. A. (1889). Z. Phys. Chem..

[cit9] Wang Y., Chen W., Zhao Q., Jin G., Xue Z., Wang Y., Mu T. (2020). Phys. Chem. Chem. Phys..

[cit10] Macfarlane D. R., Forsyth M., Izgorodina E. I., Abbott A. P., Annat G., Fraser K. (2009). Phys. Chem. Chem. Phys..

[cit11] Harris K. R. (2019). J. Phys. Chem. B.

[cit12] Fraser K. J., Izgorodina E. I., Forsyth M., Scott J. L., MacFarlane D. R. (2007). Chem. Commun..

