# Supplementary material for: Synthesis and evolution of physicochemical properties of new pharmaceutically active ionic liquids – tetracainium salicylate and tetracainium ibuprofenate
Source: RSC Adv. 2022 Sep 21;12(41):26800–7. doi: 10.1039/d2ra04711j (PMC9491082; doi:10.1039/d2ra04711j)
Supplement: RA-012-D2RA04711J-s001 [file RA-012-D2RA04711J-s001.pdf]

Electronic Supplementary Material (ESI) for RSC Advances.
This journal is © The Royal Society of Chemistry 2022

Electronic supplementary information

Synthesis and evolution of physicochemical properties of new pharmaceutically active ionic liquids – tetracainium salicylate and tetracainium ibuprofenate

Maksim Rapaić[a], Jovana Panić[a*], Branislava Teofilović[b], Nevena Grujić-Letić[b], Slobodan Gadžurić[a], Milan Vraneš[a]

[a]Faculty of Sciences, Department of Chemistry, Biochemistry and Environmental Protection, University of Novi Sad, Trg Dositeja Obradovića 3, 21000 Novi Sad, Serbia

[b]Faculty of Medicine, University of Novi Sad, Hajduk Veljkova 3, 21000 Novi Sad, Serbia

[*]Corresponding Author: Tel: +381 21 485 2751; Fax: +381 21 454 065; E–mail: jovanap@dh.uns.ac.rs

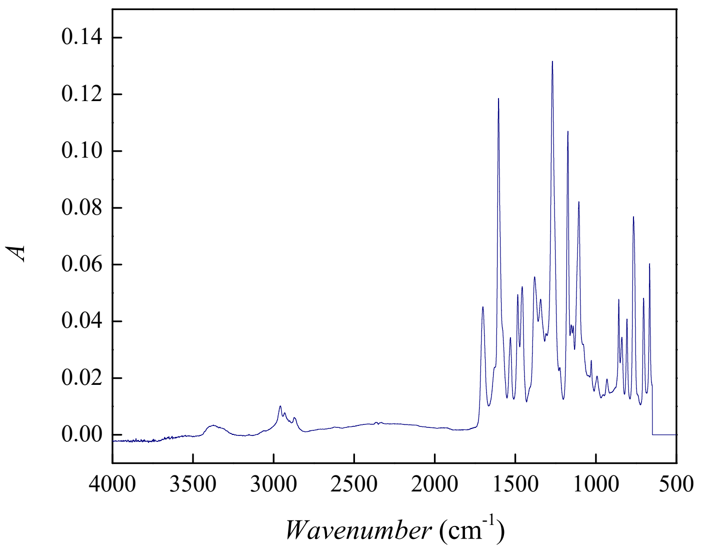

a)

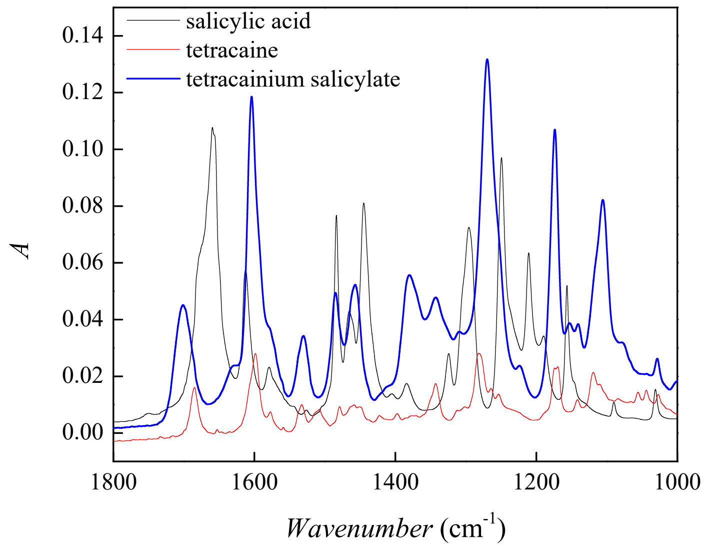

b)

Figure S1. a) IR spectrum of tetracainium salicylate; b) the IR spectra in region of 1800–1000 cm^{-1} for tetracainium salicylate, salicylic acid, and tetracaine.

IR (neat) 3500- 2700 N-H stretch and O-H stretch; 2957.86 C-H stretch; 1701.14 C=O stretch; 1603.87 conjugated C=O stretch and N-H bend; 1484.94 and 1456.94 CC stretch; 1380.06 OH in-plane bend; 1269.90 C-O stretch and C-N stretch; 1173.75 C=O strech; 766.45 stretching in the plane of the aromatic ring.

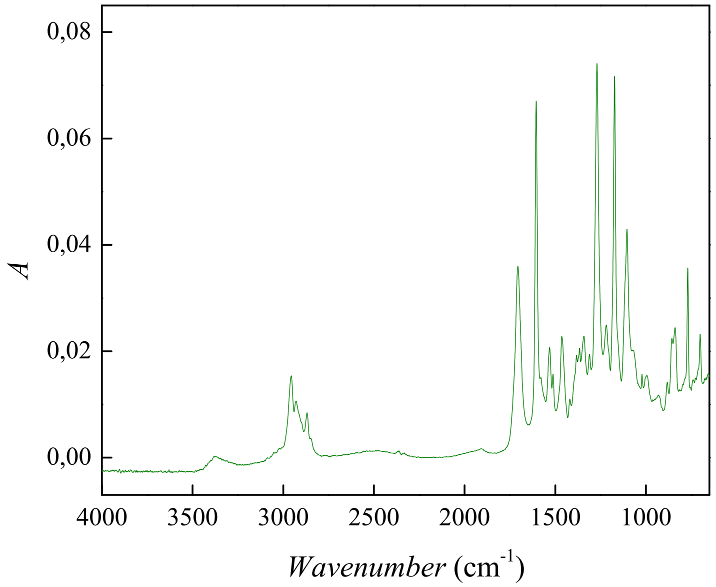

a)

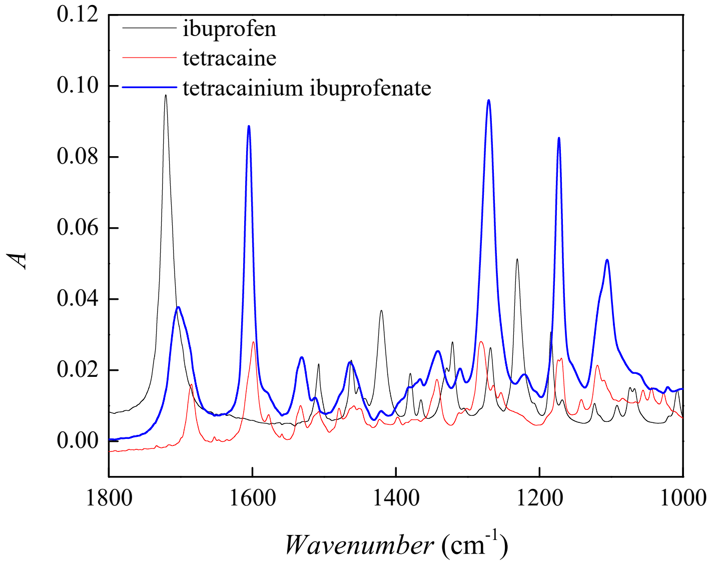

b)

Figure S2. a) IR spectrum of tetracainium ibuprofenate; b) the IR spectra in region of 1800–1000 cm^{-1} for tetracainium ibuprofenate, ibuprofen, and tetracaine.

IR (neat) 3500- 2700 N-H stretch; 2956.59 C-H stretch; 1703.27 C=O stretch; 1604.80 conjugated C=O stretch and N-H bend; 1267.19 C-O and C-N stretch; 1172.85 C=O stretch; 769.82 stretching in the plane of the aromatic ring.

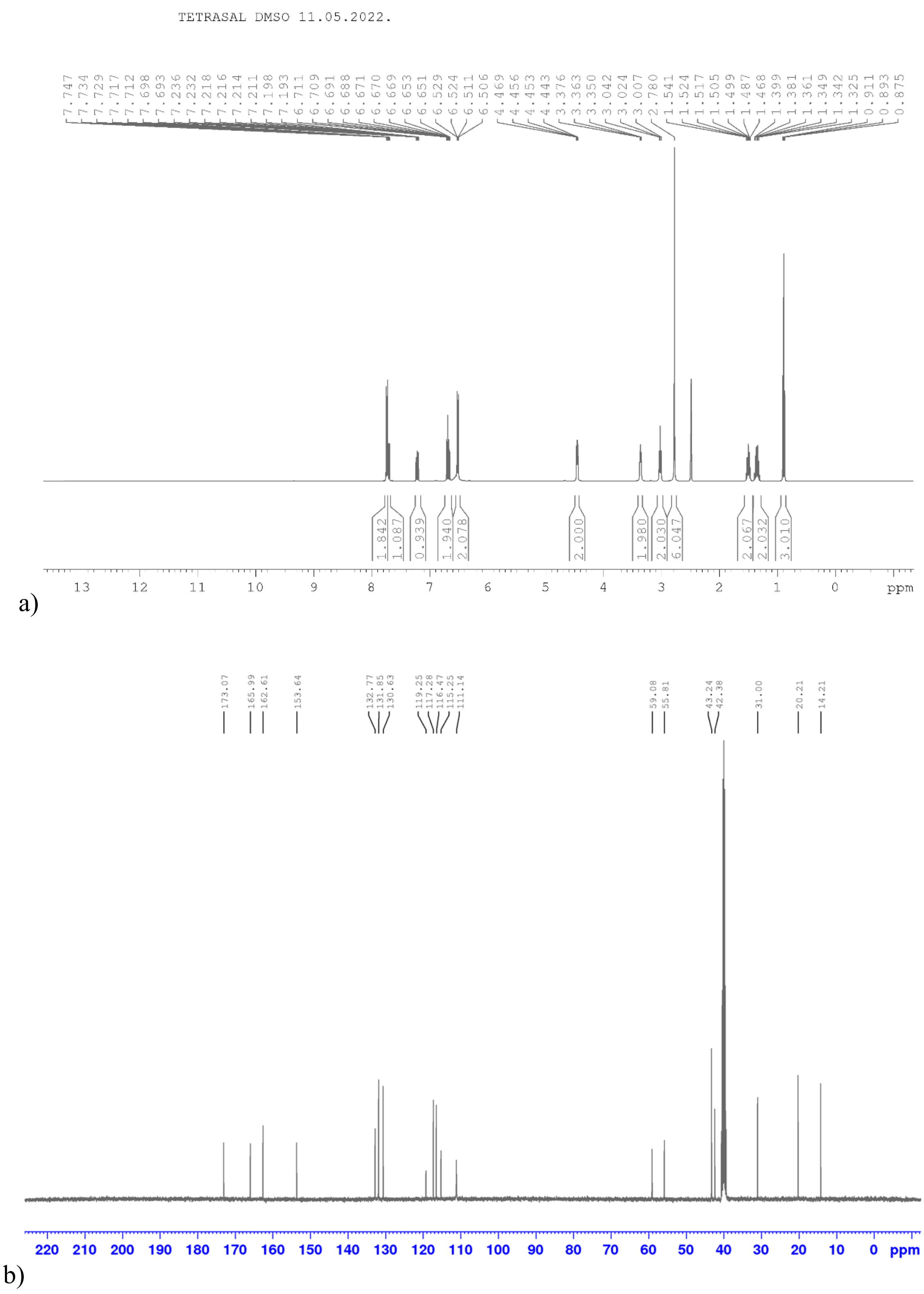

Figure S3. a)^{1}H, and b) ^{13}C NMR for synthesized tetracainium salicylate.

^{1}H NMR (400 MHz, DMSO-d6, ppm): 0.89 (t, 3H); 1.36 (m, 2H); 1.51 (m, 2H); 2.78 (s, 6H); 3.02 (t, 2H); 3.36 (t, 2H); 4.46 (t, 2H); 6.52 (d, 2H); 6.69 (m,2H); 7.22 (m, 1H); 7.71 (dd, 1H); 7.74 (d, 2H).

^{13}C NMR (101 MHz, DMSO-d6, ppm): 14.21; 20.21; 31.00; 42.38; 43.24; 55.81; 59.08; 111.14; 115.25; 116.47; 117.27; 119.25; 130.63; 131.85; 132.77; 153.61; 162.61; 165.99; 173.07.

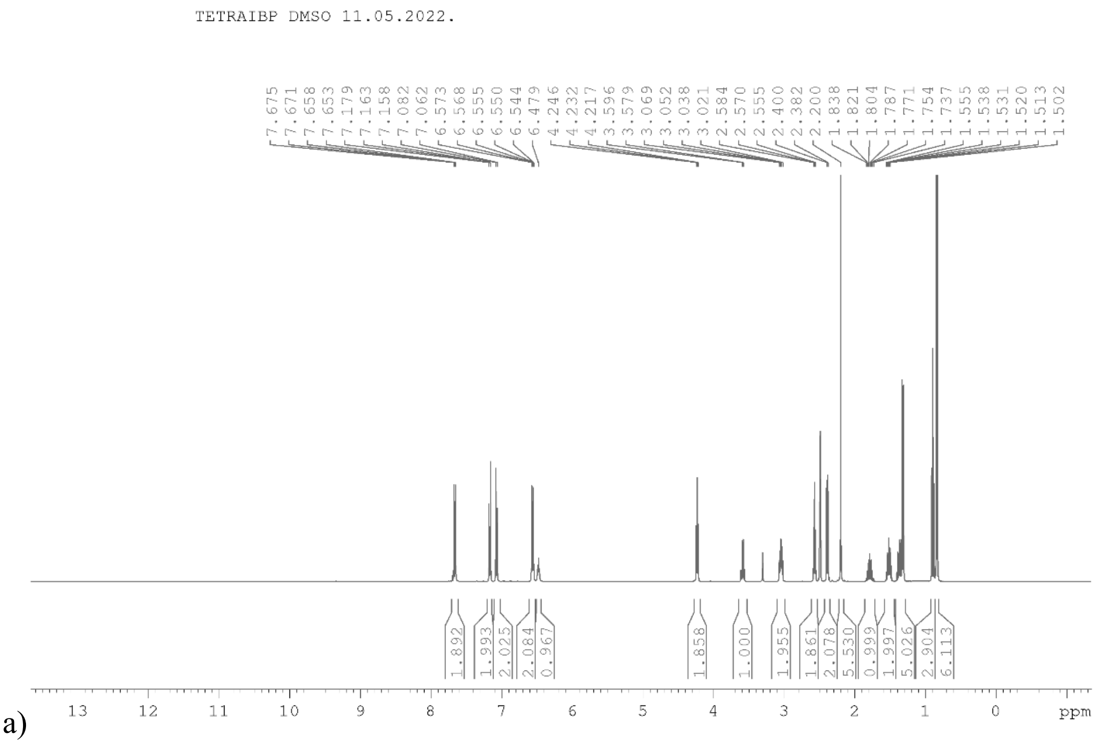

a)

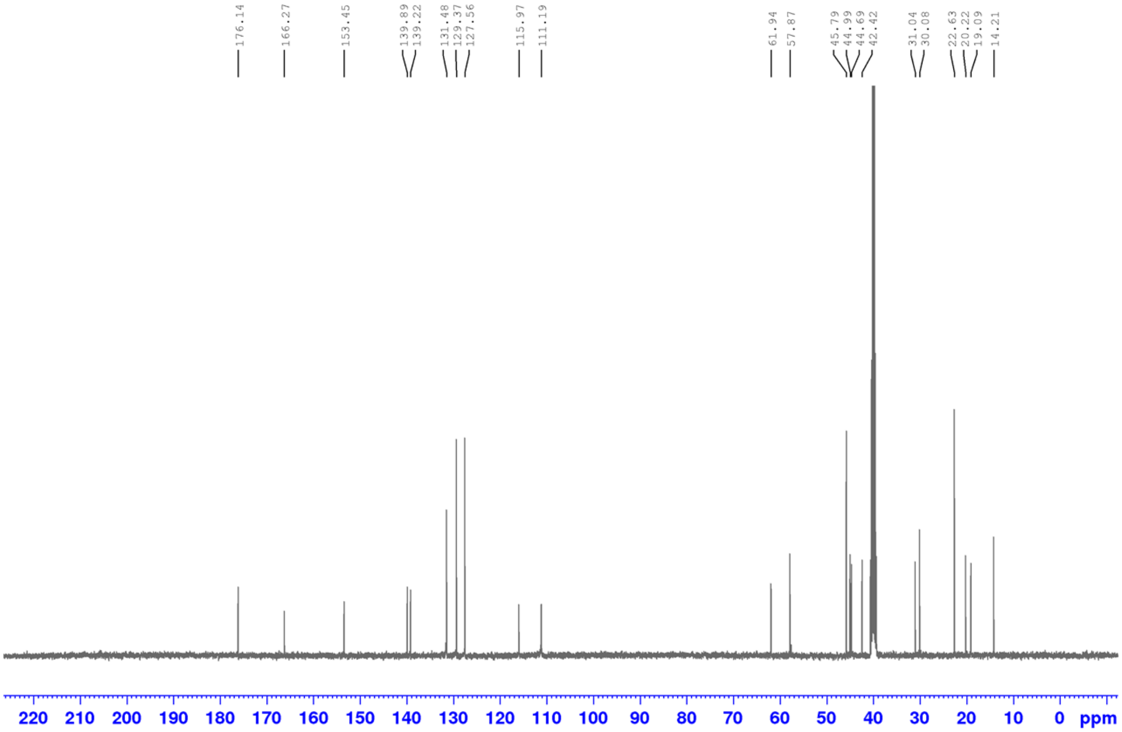

b)

Figure S4. a)^{1}H, and b) ^{13}C NMR for synthesized tetracainium ibuprofenate.

^{1}H NMR (400 MHz, DMSO-d6, ppm): 0.84 (d, 6H); 0.90 (t, 3H); 1.32 (d, 3H); 1.37 (m, 2H); 1.52 (m, 2H); 1.79 (m, 1H); 2.19 (s, 6H); 2.38 (d, 2H); 2.57 (t, 2H); 3.04 (q, 2H); 3.59 (q, 1H); 4.23 (t, 2H); 6.48 (t, 1H); 6.56 (d, 2H); 7.07 (d, 2H); 7.17 (d, 2H); 7.67 (d, 2H).

^{13}C NMR (101 MHz, DMSO-d6, ppm): 14.21; 19.09; 20.22; 22.63; 30.08; 31.04; 42.42; 44.69; 44.99; 45.79; 57.87; 61.94; 111.19; 115.97; 127.56; 129.37; 131.48; 139.89; 153.45; 166.27; 176.15.

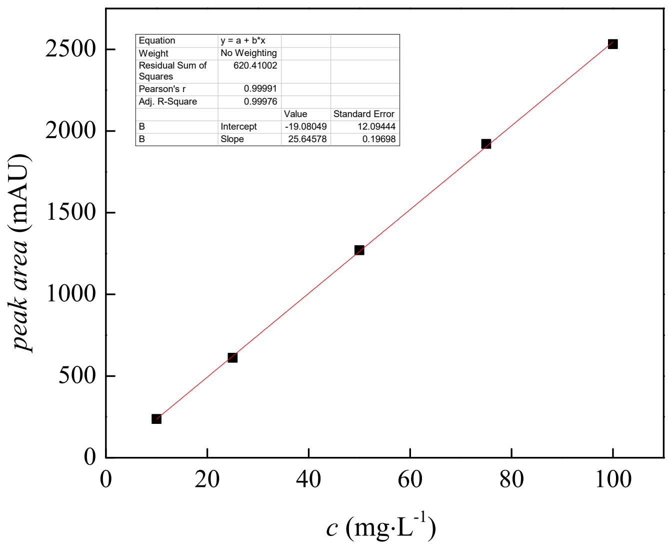

Figure S5. The calibration curve (plot of peak areas versus concentrations of tetracaine) used for the estimation purity of ionic liquids.

Table S1. Experimental values of densities (d), and thermal expansion coefficients (α_{p}) for [Tet][Sal] and [Tet][Ibp], at different temperatures.

T (K)	d (g·cm^{-3})		$\alpha_p \cdot 10^4$ (K^{-1})	
	[Tet][Sal]	[Tet][Ibp]	[Tet][Sal]	[Tet][Ibp]
293.15	/	1.04389	/	8.04
298.15	/	1.04006	/	8.07
303.15	/	1.03611	/	8.10
308.15	1.14607	1.03198	6.89	8.13
313.15	1.14235	1.02750	6.91	8.17
318.15	1.13875	1.02344	6.93	8.20
323.15	1.13516	1.01928	6.96	8.23
328.15	1.13134	1.01509	6.98	8.27
333.15	1.12725	1.01089	7.01	8.30
338.15	1.12303	1.00668	7.03	8.34
343.15	1.11879	1.00245	7.06	8.37
348.15	1.11477	0.99811	7.08	8.41

353.15	1.11072	0.99360	7.11	8.45

Table S2. Experimental values of viscosities (η) for [Tet][Sal] and [Tet][Ibp], at different temperatures.

T (K)	η (Pa·s)	
	[Tet][Sal]	[Tet][Ibp]
293.15	30655	40.430
298.15	8038.1	17.298
303.15	2411.5	8.4783
308.15	826.51	4.2498
313.15	295.17	2.2630
318.15	90.666	1.2672
323.15	38.291	0.7433
328.15	15.643	0.4835
333.15	7.6784	0.3120
338.15	4.0743	0.2100
343.15	2.2461	0.1455
348.15	1.3189	0.1028
353.15	0.8020	0.0666

Table S3. Experimental valus of electrical conductivities (κ), and calculated molar conductivities (λ_m) for [Tet][Sal] and [Tet][Ibp], at different temperatures.

T (K)	κ (μS·cm^{-1})		$\lambda_m \cdot 10^4$ (S·cm^2·mol^{-1})	
	[Tet][Sal]	[Tet][Ibp]	[Tet][Sal]	[Tet][Ibp]
318.15	1.90	1.89	6.73	8.69
323.15	2.63	2.33	9.32	10.7
328.15	3.81	2.98	13.5	13.8
333.15	6.25	3.59	22.3	16.7
338.15	8.21	4.28	29.4	20.0
343.15	12.3	5.09	44.1	23.9
348.15	17.4	6.11	62.7	28.8
353.15	25.7	7.20	93.1	34.1
